# Venous cannula occlusion during cardiopulmonary bypass recognized by ultrasonography of the internal jugular vein

**DOI:** 10.1186/s40981-022-00519-2

**Published:** 2022-04-12

**Authors:** Soshi Narasaki, Hirotsugu Miyoshi, Ryuji Nakamura, Ayako Sumii, Tomoyuki Watanabe, Sachiko Otsuki, Yasuo M. Tsutsumi

**Affiliations:** Department of Anesthesiology and Critical Care, Hiroshima University, 1-2-3 Kasumi, Minami-ku, Hiroshima, 734-8551 Japan

**Keywords:** Cervical ultrasonography, Central venous pressure, Superior vena cava occlusion, Cerebral congestion

## Abstract

**Background:**

Occlusion or malposition of the venous cannula during cardiopulmonary bypass (CPB) increases central venous pressure (CVP). When high CVP is measured, we need to determine if it is actually high or if it is measured due to catheter occlusion or technical problems with the measurement.

**Case presentation:**

We experienced a case of excessively high CVP due to malposition of the venous cannula during CPB. A 78-year-old woman underwent an aortic arch replacement for acute aortic dissection. During CPB, CVP increased up to 78 mmHg, and the time above 50 mmHg was 48 min. In this case, ultrasonography of the internal jugular vein (IJV) was useful to confirm high CVP.

**Conclusions:**

Ultrasonography is now a familiar diagnostic tool and can be used at any time. We should consider ultrasonography as the first choice for diagnosing the cause of high CVP during CPB.

## Introduction

In open-heart surgery with cardiopulmonary bypass (CPB), venous return cannulas are placed in the superior and inferior venae cavae as a standard procedure. Occlusion of the venous return cannula is a problem encountered during CPB and leads to an increase in the central venous pressure (CVP) due to the delayed return of blood to the extracorporeal circuit. In particular, occlusion of the superior vena cava (SVC) cannula can increase intracranial pressure (ICP) and decrease cerebral perfusion pressure (CPP) [1]. To avoid permanent central nervous system complications, the cause of elevated CVP must be identified and promptly corrected.

Elevated CVP during CPB may be caused by venous return cannula obstruction or technical problems with CVP measurement, such as impingement of the tip of the CVP measurement catheter in the vessel wall. The CVP increases in the former case, but not in the latter. The circuit flow rate of CPB and occlusion of the venous return catheter are commonly checked to diagnose the cause of elevated CVP. There are no standard management guidelines for troubleshooting high CVP measurements during CPB.

Herein, we report the case of a highly elevated CVP during aortic valve replacement. In this case, ultrasonography of the internal jugular vein (IJV) helped to objectively confirm the CVP.

## Case report

**Fig. 1:**
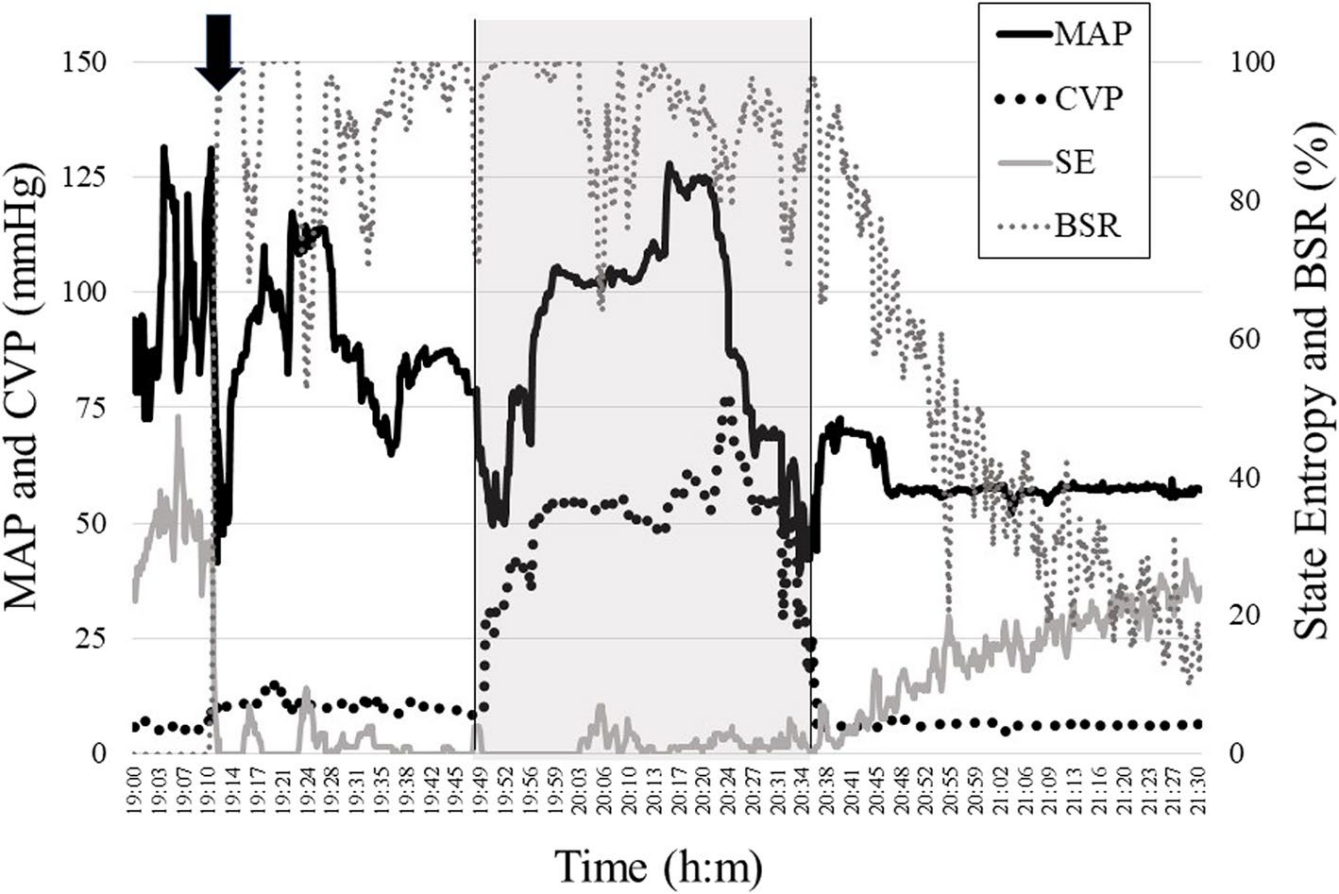
Changes in hemodynamics, state entropy, and BSR. The black line indicates the mean atrial pressure (MAP). The black dotted line represents the central venous pressure (CVP). The gray line indicates the state entropy. The gray dotted line represents the burst suppression ratio (BSR). The black arrow indicates the administration of thiopental. The gray area indicates the time during which cerebral congestion occurred. The entropy value increased gradually after the release of cerebral congestion

**Fig. 2:**
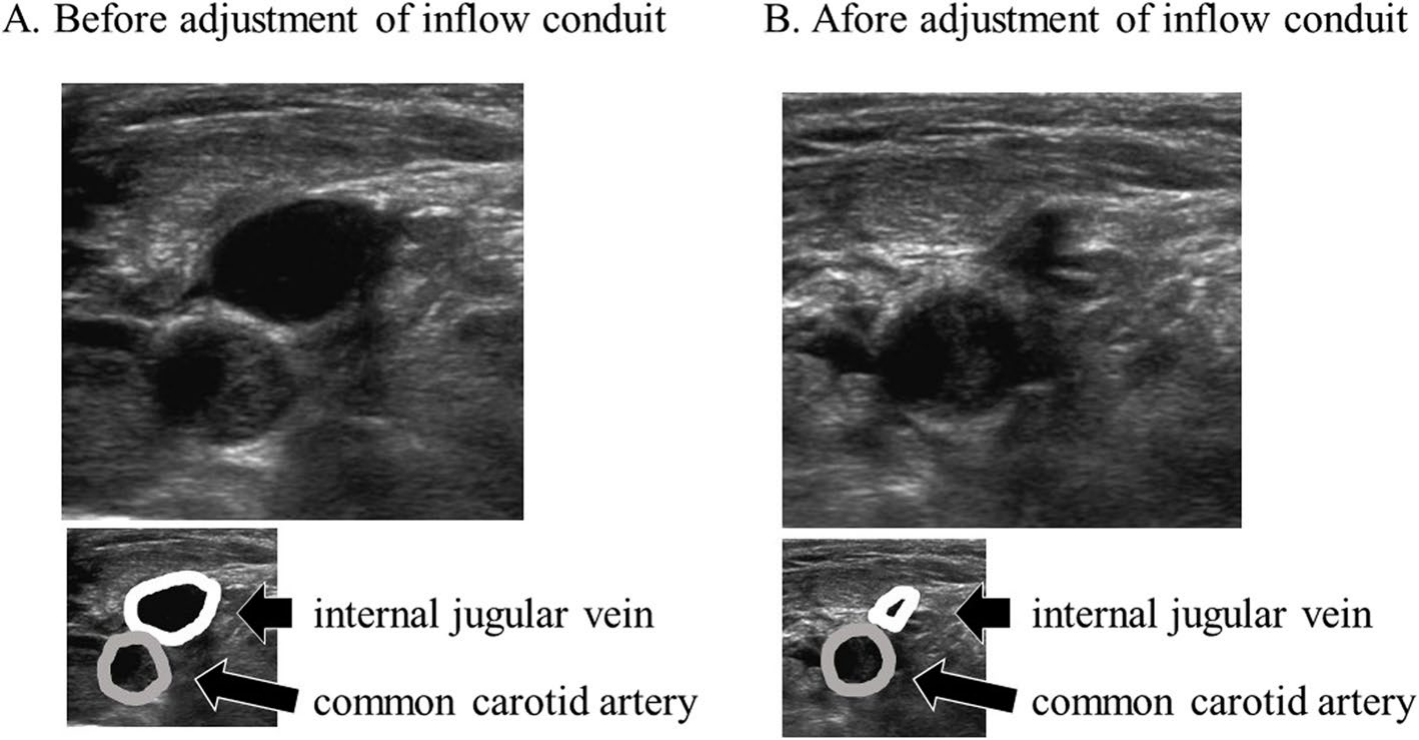
Ultrasound image of the internal jugular vein. **A** Dilated internal jugular vein (IJV) during cardiopulmonary bypass with a venous cannula in the superior vena cava. **B** Collapsed IJV after repositioning the venous cannula. We confirmed IJV distension using compression by cervical echo. Before the devascularization was corrected, the IJV had a circular shape similar to that of an artery. In addition, the IJV did not collapse upon compression by the echo probe. After the devascularization position was corrected, the IJV easily collapsed under compression

A 78-year-old woman was admitted due to impaired consciousness and was diagnosed with type A aortic dissection using computed tomography. Aortic arch replacement was performed under general anesthesia with propofol and remifentanil. We used arterial pressure measurements and central venous pressure measurements with a central venous catheter located in the right internal jugular vein and transesophageal echocardiography (TEE) to monitor hemodynamics. After heparinization, CPB was initiated by removing the blood from the superior and inferior venae cavae and returning it through the right axillary artery. A roller pump was used for selective cerebral perfusion. After reducing the body temperature to 32 °C, the superior and inferior venae cavae were clamped, and complete extracorporeal circulation was initiated. The CVP increased from 10 to 40 mmHg after the start of complete extracorporeal circulation; however, there was no change in the CPB perfusion. The anesthesiologist and surgeon on site confirmed that the inflow conduit in the surgical field was inserted into the superior vena cava; however, the CVP value on the monitor was still high. Therefore, we concluded that the CVP measurement was incorrect because of the obstruction of the central venous catheter. With increasing CVP, the mean arterial pressure increased from 75 to 120 mmHg. Epistaxis, facial edema, and blood reflux from the venous infusion line in the upper limb were observed. Cervical echography revealed IJV distention, and we concluded that the elevated CVP was due to poor blood removal. We requested the surgeon to adjust the position of the catheter tip because the elevated CVP was caused by the insertion of the inflow conduit into the azygous vein. After the adjustment of the inflow conduit, the CVP was maintained at approximately 5 mmHg. During SVC clamping, the CVP was maintained at > 50 mmHg for 48 min, with a maximum of 78 mmHg. The Entropy™ (GE Healthcare, Helsinki, Finland) value was 0 due to thiopental administration for cerebral protection before the start of CPB but increased gradually after the release of cerebral congestion. Postoperatively, the patient was observed to have poor consciousness, despite no apparent abnormal findings on postoperative computed tomography of the head. The level of consciousness gradually improved, and the patient was extubated 6 days after the surgery. The patient was moved from the intensive care unit to the general ward on the 8th postoperative day and was discharged on the 45th postoperative day without any neurological sequelae. The intraoperative course is shown in Fig. 1. Cervical ultrasonography images before and after adjusting the position of the inflow conduit are shown in Fig. 2.

## Discussion

We experienced a case of cardiovascular surgery with CVP ≥ 50 mmHg for 48 min, with a maximum of 78 mmHg. CVP is often elevated during CPB. This is frequently not recognized as a serious problem because it is often caused by impingement of the tip of the CVP measurement catheter in the vessel wall. In our patient, we could not recognize that the cause of the high CVP was obstruction of the venous cannula because the flow rate of the CPB did not decrease when the CVP increased; however, we were able to confirm this using ultrasonography.

Ultrasonography is now a very familiar diagnostic tool and is commonly used to guide nerve blocks, place catheters, and diagnose all types of lesions. An attempt has been made to estimate the CVP from the end-expiratory diameter of the IJV and the proportion of the cross-surface areas of the IJV and common carotid artery measured using ultrasonography [2, 3]. Even during CPB, it is possible to infer CVP from the properties of the IJV. In our patient, ultrasonography showed that the IJV was tense and did not collapse even when compressed with the probe, confirming that the CVP was elevated. Ultrasonography was useful in objectively determining that the CVP was actually high when a high CVP was measured using a pressure transducer.

TEE is commonly used as one of the tools to monitor intraoperative hemodynamics for both cardiac and noncardiac surgery [4, 5]. Though the azygos veins usually cannot be identified by transthoracic echocardiography, they can be identified by TEE [6]. However, the confluence between the azygos veins and the SCV can be included in the blind zone of TEE, preventing observation of the confluence with TEE. That is, an unexpected inflow conduit into an azygos vein, as in our case, could not be observed by TEE; therefore, alternative methods are warranted. While the observation of the IJV by cervical echocardiography cannot directly diagnose the misinsertion of inflow conduits into the azygos vein, it is possible to indirectly detect abnormal blood removal states by evaluating the tension of the IJV. Observation of IJV by cervical echocardiography can be useful in differentiating false high CVP due to obstruction of the central venous catheter from actual high CVP due to poor blood removal if CVP elevates during cardiopulmonary bypass.

Our patient had no neurological sequelae despite prolonged exposure to a very high CVP. CPP is determined by the mean arterial pressure minus the ICP. When the SVC cannula is occluded, as in this case, the ICP may increase and the CPP may decrease. In an experiment in pigs, Tovedal et al. reduced the flow rate of the SVC cannula by 75% during CPB and then performed pressor treatment or release of obstruction. CPP decreased after SVC cannula occlusion and recovered after both treatments [1]. In our patient, the regional cerebral oxygen saturation (rSO_2_) did not decrease during SVC cannula occlusion. Additionally, no neurological sequelae remained, although the ICP may have been elevated, as inferred from signs of congestion, such as nasal bleeding. This may be due to the fact that reflux by the roller pump increased the arterial pressure as the CVP increased and CPP was maintained. In other words, CVP elevation with a rise in blood pressure during CPB may indicate that the CVP is actually rising due to central venous congestion.

In conclusion, we encountered a case in which obstruction of the venous cannula was recognized by IJV ultrasonography. Ultrasonography can be useful as a tool to determine the cause of elevated CVP.

## Data Availability

The data used in this case report are available from the corresponding author on reasonable request.

## Abbreviations

SVC: Superior vena cava
CVP: Central venous pressure
IJV: Internal jugular vein
CPB: Cardiopulmonary bypass
ICP: Increase intracranial pressure
CPP: Cerebral perfusion pressure
TEE: Transesophageal echocardiography
